# Plant essential oil supplementation promotes growth and attenuates lipopolysaccharide (LPS)-induced acute liver injury through SIRT1/PGC-1α signaling pathway in nursery pigs

**DOI:** 10.5713/ab.25.0066

**Published:** 2025-06-04

**Authors:** Yu Niu, Xinru Song, Yiying Chen, Yiting Xu, Yiru Chen, Qingzhou Lin, Jintian He, Jinsong Liu, Ruiqiang Zhang, Caimei Yang

**Affiliations:** 1 College of Animal Science and Technology·College of Veterinary Medicine, Zhejiang A and F University, Hangzhou, China; 2 Zhejiang Vegamax Biotechnology Co., Ltd., Huzhou, China

**Keywords:** Growth Performance, Oxidative Stress, Piglets, Plant Essential Oil, Signaling Pathway

## Abstract

**Objective:**

This study aimed to evaluate whether dietary supplementation with plant essential oil (PEO) and coated plant essential oil (CEO) could promote growth and alleviate liver oxidative damage in nursery piglets challenged with lipopolysaccharide (LPS) by modulating mitochondrial function in the liver.

**Methods:**

Twenty-four 21-day-old piglets were randomly assigned to four groups, with six replicates per group. The CON and LPS groups received a basal diet, while the LPS+PEO and LPS+CEO groups were received the basal diet supplemented with 500 mg/kg of PEO and 500 mg/kg of CEO, respectively. The experimental period lasted for 28 days. On day 49, piglets in the LPS, LPS+PEO, and LPS+CEO groups were injected intraperitoneally with LPS at a dose of 100 μg/kg body weight, while those in the CON group received an equal volume of saline. All piglets were weighed and euthanized four hours after the LPS or saline injection. Blood and liver samples were collected for further analysis.

**Results:**

Piglets in the LPS+PEO and LPS+CEO groups showed higher (p<0.05) average daily gain and better feed conversion ratio, and increased mRNA expressions of liver *HO-1*, *NQO1* and *Trx2* compared to the LPS and CON groups. Diet supplemented with PEO and CEO increased (p<0.05) the contents of immunoglobulin A (IgA), immunoglobulin G and immunoglobulin M (IgM), and the protein expressions of SIRT1 and PGC-1α in the liver of LPS-induced nursery piglets. Furthermore, piglets in the LPS+CEO group exhibited higher (p<0.05) levels of IgA, IgM, total antioxidant capacity, and the mRNA expressions of *SOD2* and *Trx2* in the liver than those of the LPS+PEO group.

**Conclusion:**

Dietary supplementation with PEO or CEO improved growth performance in nursery piglets and alleviated LPS-induced liver oxidative damage in nursery piglets through activation of the SIRT1/PGC-1α signaling pathway. In addition, CEO supplementation demonstrated a more pronounced antioxidant effect than PEO.

## INTRODUCTION

Weaned piglets are highly susceptible to oxidative stress due to a combination of abrupt dietary changes, immature antioxidant systems, gut microbiota dysbiosis, and stress-induced inflammation during the post-weaning period. The sudden transition from antioxidant-rich sow’s milk to solid feed reduces antioxidant intake, while their underdeveloped endogenous antioxidant systems are unable to neutralize excessive reactive oxygen species (ROS). Simultaneously, diet-induced gut microbiota dysbiosis promotes the proliferation of pathogenic bacteria, increasing the release of lipopolysaccharide (LPS), a toxin that exacerbates intestinal inflammation and permeability [1,2]. Additional stressors, such as social separation and environmental changes, elevate cortisol levels, further intensifying inflammation and ROS production, ultimately leading to oxidative stress in weaned piglets. Oxidative stress can damage mitochondrial DNA, disrupt protein structure, and impair mitochondrial and liver function [3]. Activating the SIRT1/PGC-1α pathway promotes mitochondrial biosynthesis, enhances energy metabolism, and combats oxidative stress. Oxidative stress also weakens hepatic antioxidant capacity, disrupts nutrient metabolism, and hinders animal growth, highlighting the importance of nutritional liver regulation in weaned piglets.

Plant essential oils (PEOs), aromatic secondary metabolites from plants, contain terpenoids and aromatic compounds with antibacterial, anti-inflammatory, antiviral, antioxidant, and immunomodulatory properties [4]. Recognized for their antioxidant potential, PEOs have become prominent feed additives. Thymol supplementation increased hepatic superoxide dismutase (SOD) and glutathione peroxidase (GSH-Px) activity in grass carp [5], while carvacrol-thymol mixtures improved jejunal antioxidant enzyme levels and reduced ROS in piglets [6]. *Cistus* essential oils also repaired DNA damage and promoted mitochondrial biogenesis via SIRT1 and PGC-1α activation [7].

However, PEOs are volatile and prone to degradation from light, oxygen, and heat. Encapsulation offers protection by limiting oxygen exposure, reducing oxidation-related efficacy loss, and enhancing bioavailability and delivery efficiency [8–10]. Hydrophobic essential oils can form inclusion complexes with cyclodextrins, enhancing their water solubility and absorption in the body. This coating also protects the essential oils from the harsh gastrointestinal environment, enabling a gradual release in the small intestine where absorption occurs. Such controlled release markedly enhances bioavailability and maximizes the physiological benefits of essential oils [11]. Studies have shown that cinnamaldehyde nanoemulsion improves antioxidant enzyme activity and enhances both immunity and antioxidant status in Nile tilapia [12]. Likewise, nanoemulsions of tarragon essential oil demonstrated superior free radical scavenging and antioxidant capacity compared to uncoated forms [13].

Despite these findings, few studies have directly compared the effects of PEO and coated plant essential oils (CEO) on hepatic antioxidant activity in weaned piglets or explored their underlying mechanisms. Additionally, LPS is known to induce ROS production, leading to acute oxidative stress and mitochondrial dysfunction in hepatocytes [14]. Thus, in this study, an acute liver oxidative damage model was established by intraperitoneal injection of *Escherichia coli* LPS. The objective was to investigate whether dietary supplementation with PEO and CEO could promote growth performance in nursery piglets and attenuate liver oxidative damage of nursery piglets challenged with LPS. The findings aim to provide a theoretical foundation for the effective application of essential oils in piglet nutrition.

## MATERIALS AND METHODS

### Preparation of plant essential oil and coated plant essential oil

The PEO and CEO used in this study were supplied by Zhejiang Vegamax Biotechnology. Both PEO and CEO primarily consisted of 27% cinnamaldehyde, 9% thymol, and 4% vanillin. The remaining 60% comprised high-molecular-weight coating materials, including seaweed polysaccharides and porous starch.

### Animals, diets and experimental design

A total of 24 nursery piglets ([Duroc×Landrace]×Large White; equal numbers of males and females), aged 21 days, were weaned and randomly assigned to four treatment groups, with six piglets per pen. The experiment lasted for 28 days. Piglets in the CON and LPS groups received a basal diet, while those in the LPS+PEO and LPS+CEO groups were fed the basal diet supplemented with 500 mg/kg of PEO and 500 mg/kg of CEO, respectively. The trial was conducted at Chia Tai Pig Industry, and all piglets had *ad libitum* access to feed and water throughout the experimental period. The composition and nutrient content of the basal diet for nursery piglets are presented in Table 1.

### Sampling and preparation

On day 28 of the trial, piglets in the LPS, LPS+PEO, and LPS+CEO groups were weighed and injected intraperitoneally with *E. coli* LPS (strain O55:B5) at a dose of 100 μg/kg body weight. Piglets in the CON group received an equivalent volume of sterile saline. Four hours after the injections, all piglets were weighed again and blood samples were drawn into 10 mL procoagulant tubes collected via anterior vena cava puncture. Then all piglets were euthanized via intravenous administration of 30 mg/kg pentobarbital and exsanguinated after deep anesthesia. The abdominal cavity of each piglet was opened using a scalpel, and liver tissues were excised and weighed. Approximately 0.1 g of liver samples were placed into 2 mL transparent polypropylene cryogenic tubes, then stored at −80°C for subsequent use.

### Growth performance

The body weight of each piglet was recorded at 21 and 49 days of age, prior to the LPS injection. Daily feed intake for each group was monitored over a 28-day period. At the end of the experiment, the average daily gain (ADG), average daily feed intake (ADFI), and feed conversion ratio (FCR) were calculated.

### Liver injury index

The liver was gently lifted to separate the remaining connective tissues, then carefully excised and weighed to determine the liver index. The liver index was calculated using the following formula:


(1) Liver index (g/kg)=Liver weight (g)/Live weight before slaughter (kg)

### Liver injury markers analysis

Serum was isolated from blood samples via centrifugation at 3,500×g for 10 min at 4°C. The activities of serum alanine aminotransferase (ALT) and aspartate aminotransferase (AST) were measured according to the manufacturer’s instructions provided with the kits, which were purchased from Nanjing Jiancheng Bioengineering Research Institute.

### Liver immune function analysis

Liver samples were homogenized in saline (wt: vol = 1: 9) using a homogenizer and then centrifuged at 4°C at 3,000×g for 10 min. The supernatant was collected for the measurement of immune and antioxidant indices. The contents of immunoglobulin A (IgA), immunoglobulin G (IgG), immunoglobulin M (IgM), interleukin-1β (IL-1β), interleukin-6 (IL-6), and tumor necrosis factor-α (TNF-α) in the liver were quantified using enzyme-linked immunosorbent assay (ELISA). The assays were conducted using kits from Nanjing Angle Gene Biotechnology.

### Liver antioxidant index analysis

The activities of total antioxidant capacity (T-AOC), catalase (CAT), total superoxide dismutase (T-SOD), GSH-Px, and the concentration of malondialdehyde (MDA) in the liver were measured using kits from Nanjing Jiancheng Bioengineering Research Institute, following the manufacturer’s instructions.

### Quantitative real-time polymerase chain reaction analysis

Total RNA from the liver was extracted following the manufacturer’s instructions for RNAiso Plus reagent (Takara Biomedical Technology). RNA concentration was measured using a Nano-300 microspectrophotometer. The concentration was recorded and adjusted to 500 ng/μL. A 1 μL aliquot of total RNA was reverse transcribed into cDNA using a polymerase chain reaction (PCR) instrument, in accordance with the instructions of the PrimeScript RT Reagent Kit (Takara) with gDNA Eraser. The primers used are shown in Table 2. Quantitative PCR was performed on a CFX96 fluorescence quantitative PCR instrument, following the instructions of the TB Premix EX Taq II (Takara). The reaction conditions were as follows: pre-denaturation at 95°C for 30 s, followed by 40 cycles of denaturation at 95°C for 5 s, and annealing/extension at 60°C for 30 s. Relative mRNA expression in the liver was calculated using the 2^−ΔΔCT^ method, with β-actin serving as the internal reference gene.

### Western blot analysis

Liver samples were homogenized using lysate (PMSF:NP-40 = 1:99) and centrifuged at 4°C at 16,000×g for 10 min to obtain the supernatant. Protein concentration was quantified using a BCA kit (Beyotime). After heat denaturation, proteins were separated by sodium dodecyl sulfate-polyacrylamide gel electrophoresis (SDS-PAGE), and the separated protein bands were transferred to PVDF membranes. Following transfer, the membranes were blocked with 5% skimmed milk for 2 h. The membranes were then incubated overnight at 4°C with rabbit anti-PGC-1α monoclonal antibody (dilution 1:500; ABclonal), rabbit anti-SIRT1 monoclonal antibody (dilution 1:800; Abways), and rabbit anti-β-actin monoclonal antibody (dilution 1:5,000; Proteintech), respectively. Afterward, the membranes were incubated at room temperature for 1 h with horseradish peroxidase-conjugated anti-rabbit IgG (dilution 1:1,000; Beyotime). The signals of the target proteins were detected using a fluorescence imager (LAS-4000), and the grayscale values of the protein bands were analyzed using Gel Pro Analyzer 4.0 software. The relative expression of the target proteins was determined by calculating the ratio of the grayscale values of the SIRT1 and PGC-1α bands to that of the internal reference protein β-actin.

### Statistical analysis

All data were statistically analyzed using SPSS 26.0 software (IBM). Each piglet was considered an experimental unit. Data were evaluated using one-way analysis of variance (ANOVA), followed by Duncan’s multiple range test for post hoc comparisons. Results are presented as mean±SEM, and differences were considered statistically significant at p<0.05.

## RESULTS

### Growth performance

As shown in Table 3, diets supplemented with PEO and CEO significantly increased (p<0.05) final body weight, ADG, and ADFI, while significantly improving (p<0.05) FCR in nursery piglets compared to the CON and LPS groups. Furthermore, piglets in the LPS+CEO group exhibited significantly higher (p<0.05) final body weight and ADG, and better (p<0.05) FCR than those in the LPS+PEO group.

### The liver index

**Figure 1:**
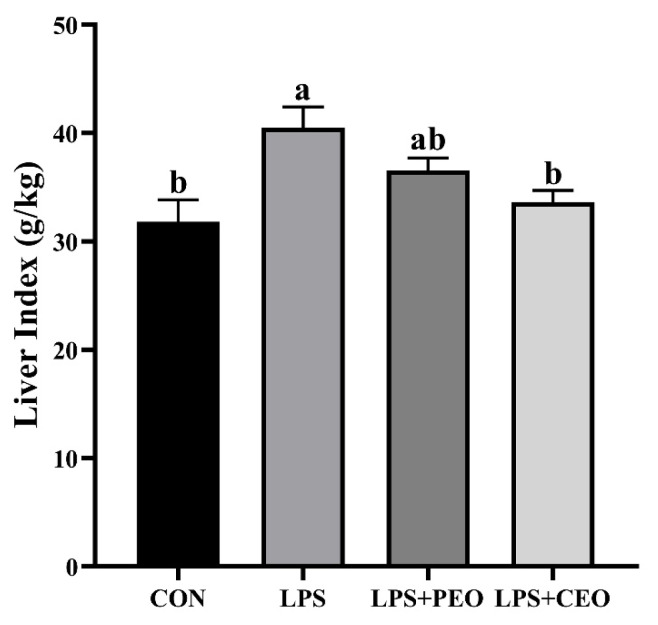
The effect of dietary supplementation with plant essential oil (PEO) and coated plant essential oil (CEO) on the liver index of nursery piglets challenged by lipopolysaccharide (LPS). CON, control group, piglets fed the basal diet and injected with 100 μg/kg BW saline; LPS, piglets fed the basal diet and injected with LPS at a dose of 100 μg/kg body weight; LPS+PEO, piglets fed the basal diet supplemented with 500 mg/kg PEO and injected with LPS; LPS+CEO, piglets fed the basal diet supplemented with 500 mg/kg CEO and injected with LPS. ^a,b^ Means with different superscript differ significantly (p<0.05).

As shown in Figure 1, LPS significantly increased (p<0.05) the liver index compared to the CON group. However, among the LPS-challenged piglets, dietary supplementation with CEO significantly reduced (p<0.05) the liver index.

### The liver function indicators in the serum

**Figure 2:**
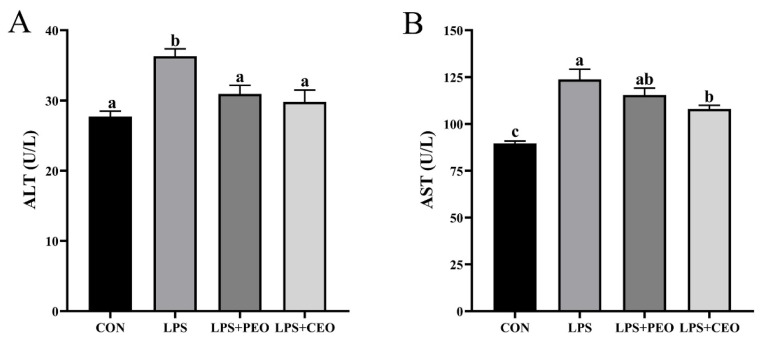
The effect of dietary supplementation with plant essential oil (PEO) and coated plant essential oil (CEO) on the activities of alanine aminotransferase (ALT) and aspartate aminotransferase (AST) in the serum of nursery piglets challenged by lipopolysaccharide (LPS). CON, control group, piglets fed the basal diet and injected with 100 μg/kg BW saline; LPS, piglets fed the basal diet and injected with LPS at a dose of 100 μg/kg body weight; LPS+PEO, piglets fed the basal diet supplemented with 500 mg/kg PEO and injected with LPS; LPS+CEO, piglets fed the basal diet supplemented with 500 mg/kg CEO and injected with LPS. ^a–c^ Means with different superscript differ significantly (p<0.05).

As shown in Figure 2, LPS significantly increased (p<0.05) the activities of ALT and AST compared to the CON group. In contrast, dietary supplementation with PEO significantly reduced (p<0.05) ALT activity in LPS-challenged piglets. Moreover, CEO supplementation significantly decreased (p<0.05) both ALT and AST activities compared to the LPS group.

### The liver immune function

As presented in Table 4, LPS significantly reduced (p<0.05) the contents of IgA, IgG and IgM in the liver of nursery piglets, and significantly increased (p<0.05) the contents of IL-1β, IL-6 and TNF-α compared to CON group. In contrast, diet supplemented with PEO significantly increased (p<0.05) the contents of IgA and IgM in the liver of LPS-challenged nursery piglets. Dietary CEO supplementation showed increased (p<0.05) the contents of IgA, IgG and IgM in the liver of nursery piglets and decreased (p<0.05) the contents of IL-1β, TNF-α and IL-6 than piglets in LPS group. Moreover, LPS+ CEO group showed higher (p<0.05) concentrations of IgA and IgM than those of LPS+PEO group. As shown in Table 4, LPS significantly reduced (p<0.05) the hepatic contents of IgA, IgG, and IgM, and significantly increased (p<0.05) the levels of IL-1β, IL-6, and TNF-α compared to the CON group. In contrast, dietary supplementation with PEO significantly increased (p<0.05) the hepatic levels of IgA and IgM in LPS-challenged nursery piglets. Moreover, CEO supplementation significantly increased (p<0.05) the hepatic contents of IgA, IgG, and IgM, and decreased (p<0.05) the levels of IL-1β, TNF-α, and IL-6 compared to the LPS group. Piglets in the LPS+CEO group had significantly higher (p<0.05) concentrations of IgA and IgM than those in the LPS+PEO group.

### The liver antioxidant index

As shown in Table 4, LPS significantly decreased (p<0.05) the hepatic activities of T-SOD, T-AOC, GSH-Px, and CAT, and significantly increased (p<0.05) the concentration of MDA in the liver of piglets compared to the CON group. In contrast, dietary supplementation with CEO significantly increased (p<0.05) the activities of T-SOD, T-AOC, GSH-Px, and CAT, and significantly reduced (p<0.05) the concentration of MDA compared to the LPS group. Furthermore, the T-AOC level in the LPS+CEO group was significantly higher (p<0.05) than that in the LPS+PEO group.

### The relative mRNA expression levels of genes in the liver

As shown in Table 5, LPS significantly downregulated (p< 0.05) the mRNA expression levels of *Keap1*, *HO-1*, *SOD1*, *SOD2*, and *CAT* compared to the CON group. In contrast, dietary supplementation with PEO significantly upregulated (p<0.05) the mRNA expression of *HO-1* and *NQO1* in LPS-challenged piglets. Moreover, supplementation with CEO significantly upregulated (p<0.05) the mRNA expression of *Nrf2*, *HO-1*, *NQO1*, *SOD2*, and *CAT* compared with the LPS group. The LPS+CEO group also showed higher (p<0.05) mRNA expression of *SOD2* than the LPS+PEO group. These antioxidant-related genes were further analyzed by Principal Component Analysis, which revealed that *NQO1* (PC1 = 0.638; PC2 = −0.577) and *HO-1* (PC1 = 0.600; PC2 = −0.477) were among the top principal contributors.

In addition, LPS significantly downregulated (p<0.05) the mRNA expression of *SIRT1*, *PGC-1α*, *TFAM*, and *Trx2* compared to the CON group. In contrast, dietary supplementation with PEO significantly upregulated (p<0.05) the mRNA expression of *Prx3* and *Trx2* in LPS-challenged piglets. In addition, CEO supplementation significantly upregulated (p<0.05) the mRNA expression of *SIRT1*, *PGC-1α*, *Nrf1*, *Prx3*, *Trx-R2*, and *Trx2* compared to the LPS group. Additionally, the mRNA expression levels of *PGC-1α* and *Trx2* were significantly higher (p<0.05) in the LPS+CEO group than in the LPS+PEO group.

### The protein expression levels of mitochondrial function in the liver

**Figure 3:**
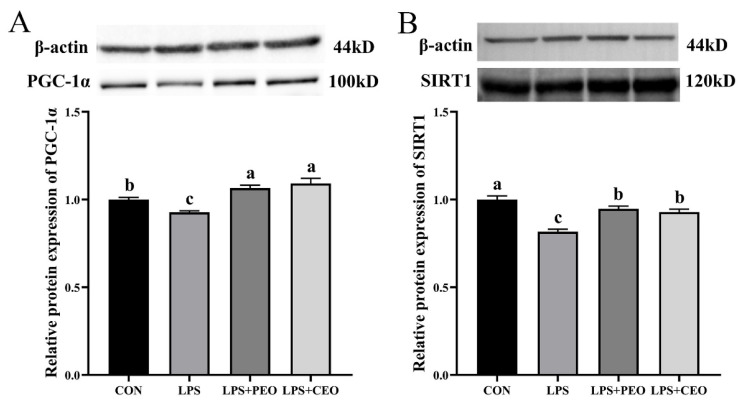
Effect of dietary supplementation with plant essential oil (PEO) and coated plant essential oil (CEO) on the protein expression levels of SIRT1/PGC-1α signaling pathway in the liver of nursery piglets challenged by lipopolysaccharide (LPS). CON, control group, piglets fed the basal diet and injected with 100 μg/kg BW saline; LPS, piglets fed the basal diet and injected with LPS at a dose of 100 μg/kg body weight; LPS+PEO, piglets fed the basal diet supplemented with 500 mg/kg PEO and injected with LPS; LPS+CEO, piglets fed the basal diet supplemented with 500 mg/kg CEO and injected with LPS. ^a–c^ Means with different superscript differ significantly (p<0.05). PGC-1α, peroxisome proliferator-activated receptor γ coactivator 1α; SIRT1, silent information regulator 1.

As shown in Figure 3, western blot analysis revealed that LPS significantly decreased (p<0.05) the relative protein expression levels of SIRT1 and PGC-1α in the liver compared to the CON group. In contrast, dietary supplementation with either PEO or CEO significantly upregulated (p<0.05) the protein expression of SIRT1 and PGC-1α compared to the LPS group.

## DISCUSSION

A previous study demonstrated that dietary supplementation with a compound of carvacrol, cinnamaldehyde, and thymol significantly increased BW and ADG and reduced FCR in piglets compared to a basal diet [15]. In the present study, dietary supplementation with PEO and CEO, both containing 27% cinnamaldehyde, 9% thymol, and 4% vanillin, significantly improved ADG, ADFI, and final BW, while improving FCR in nursery piglets compared to the CON and LPS groups. These findings may be attributed to the ability of cinnamaldehyde and thymol to enhance feed palatability, thereby promoting feed intake and improving weight gain.

The liver plays a key role in metabolism, synthesis, detoxification, and immunity, with liver index serving as an indicator of its health. In this study, LPS significantly increased the liver index in nursery pigs, suggesting liver damage, consistent with the reports that LPS-induced oxidative stress causes hepatocyte swelling, intrahepatic hemorrhage, and liver enlargement [16]. In contrast, a CEO-supplemented diet reduced the liver index, indicating potential protective effects.

Serum ALT and AST activities, common markers of liver injury, were significantly elevated in the LPS group, confirming successful model induction. Essential oil-based plant feed additives like carvacrol, thymol, and cinnamaldehyde have previously been shown to lower AST and ALT levels in broilers [17], while cinnamaldehyde reduced these enzymes in *Salmonella gallinarum*-challenged chicks and alleviated liver inflammation [18]. Similarly, PEO reduced serum ALT, and CEO reduced both ALT and AST in LPS-challenged piglets in this study, likely by inhibiting the release of hepatic enzymes into the bloodstream through tissue repair mechanism. Due to microencapsulation, CEO offers greater stability and bioavailability than PEO, enhancing its hepatoprotective effects.

Excessive free radicals produced by oxidative stress stimulate immune cells, resulting in reduced immune function, thereby releasing proinflammatory cytokines and initiating inflammatory responses. The immunoglobulins, such as IgA, IgG, and IgM, are involved in regulating inflammatory responses and eliminating pathogens. As proinflammatory cytokines, IL-6, IL-1β, and TNF-α may lead to chronic inflammatory states and disruption of biological homeostasis [19]. Adding thymol, carvacrol and eugenol to the diet can increase the IgG titer and total antibody titer of broiler chickens, produce immunostimulatory effects, and enhance humoral immune responses [20,21]. Oregano essential oil can increase the levels of IgA and IgM in the blood of calves, effectively enhance the specific immune response ability of calves, and improve the immune status [22]. Li et al [23] reported that a capsule essential oil product containing thymol and cinnamaldehyde can increase the levels of IgA and IgM in weaned piglets. Adding cinnamaldehyde to the diet can significantly reduce the levels of IL-6, IL-1β and TNF-α in serum and tissues, indicating that the diet supplemented with cinnamaldehyde can protect and reduce LPS-induced inflammation, resist the invasion of LPS, and show good anti-inflammatory function [24]. Previous research results have shown that thymol regulates the inflammatory state of weaned piglets by inhibiting the release of LPS-induced inflammatory mediators, thereby exerting an anti-inflammatory effect [25]. In the current study, supplementation with PEO and CEO significantly increased IgM and IgA levels in the liver, while CEO additionally elevated IgG and decreased IL-6, IL-1β, and TNF-α concentrations. These findings indicate that both PEO and CEO enhance humoral immunity and mitigate inflammatory responses in LPS-challenged piglets. This immunomodulatory effect is likely due to the promotion of immunoglobulin synthesis and secretion by immune cells, which subsequently suppressed the expression and release of pro-inflammatory cytokines. The encapsulated form of essential oils, CEO, owing to its improved storage stability and bioavailability, exerted more pronounced anti-inflammatory effects than the free form, PEO.

The level of T-AOC serves as a key indicator of the body’s overall ability to resist oxidative stress and reflects the antioxidant status. Antioxidant enzymes such as CAT, T-SOD, and GSH-Px play critical roles in neutralizing harmful free radicals and reducing oxidative damage, while MDA is a reliable biochemical marker for oxidative stress [26]. A previous study reported that dietary supplementation with essential oils increased the activities of CAT, T-SOD, and T-AOC, and reduced MDA concentrations in the intestines of weaning pigs challenged by K88 *E. coli*, thereby enhancing their antioxidant capacity [27]. Cinnamaldehyde was also shown to improve antioxidant status and alleviate oxidative damage in the kidneys and heart by increasing the activities of T-SOD, CAT, and GSH-Px and decreasing MDA levels [28,29]. In the present study, CEO supplementation significantly increased the activities of T-SOD, T-AOC, GSH-Px, and CAT, and decreased the hepatic MDA content in LPS-challenged nursery piglets. These findings suggested that CEO enhanced the antioxidant defense system by promoting free radical scavenging. This effect may be associated with the ability of essential oils to boost antioxidant enzyme activity, which helps to mitigate damage to the electron transport chain and supports mitochondrial oxidative phosphorylation and biofunction.

The Nrf2/HO-1 signaling pathway is essential for maintaining redox homeostasis, as it counteracts oxidative stress and prevents cellular damage. Under oxidative stress, Nrf2 translocates to the nucleus, where it promotes the transcription of antioxidant genes and activates several downstream antioxidant enzymes [30]. Among these, NQO1 and HO-1 are key Nrf2-regulated enzymes that synergistically combat oxidative stress. As such, NQO1 detoxifies quinones and stabilizes antioxidants, while HO-1 produces bilirubin and carbon monoxide, both of which reduce ROS levels and suppress inflammation [31]. Previous studies have shown that eugenol alleviated transmissible gastroenteritis virus-induced reductions in Nrf2, HO-1, and NQO1 protein expression in IPEC-J2 cells [32], and that OEO upregulated both mRNA and protein levels of nuclear Nrf2 and SOD1 in H_2_O_2_-treated IPEC-J2 cells, indicating a protective role against intestinal oxidative damage through activation of the Nrf2 pathway [33]. Consistent with these findings, our results demonstrated that dietary supplementation with both PEO and CEO significantly upregulated the hepatic mRNA expression of HO-1 and NQO1 in LPS-challenged piglets. Moreover, CEO supplementation further enhanced the expression of Nrf2, SOD2, and CAT compared to the LPS group. These results suggest that essential oils activated Nrf2 signaling, facilitating its nuclear translocation and thereby inducing the expression of downstream antioxidant genes and promoting antioxidant enzyme production. Consequently, PEO and CEO contributed to improved antioxidant capacity and maintenance of redox balance in the liver. PEOs, due to their volatile nature, are susceptible to oxidative degradation and environmental factors such as oxygen exposure, light, and thermal stress. Encapsulation technologies involving cross-linking have been shown to improve the physicochemical properties of essential oils. These technologies enhance the stability of essential oils by minimizing rapid evaporation and degradation of active components, and improve their solubility in aqueous environments, thereby increasing their bioavailability and antioxidant efficacy [8,9]. As a result, CEO exhibited a stronger protective effect against liver dysfunction than PEO.

Mitochondria are multifunctional organelles that actively regulate cellular metabolism, various cellular functions, and maintain intracellular homeostasis. The electron transport chain on the mitochondrial inner membrane generates ROS, which subsequently triggers redox reactions [34]. However, excessive ROS can lead to oxidative stress. The mitochondrial thioredoxin system, which includes Trx2, Trx-R2, and Prx3, is a crucial non-enzymatic antioxidant system responsible for regulating mitochondrial redox homeostasis. As a key regulator of antioxidant defense and mitochondrial biogenesis, PGC-1α enhances mitochondrial oxidative phosphorylation and helps maintain cellular energy metabolic homeostasis. The histone deacetylase SIRT1 activates PGC-1α through deacetylation, thereby improving mitochondrial function and promoting liver health [35]. Additionally, Nrf1, a nuclear transcription factor and downstream target of the SIRT1/PGC-1α pathway, is involved in maintaining redox balance in the liver and protecting cells from oxidative stress [36]. Previous studies have demonstrated that cinnamaldehyde [37] and carvacrol [38] upregulated the expression of PGC-1α and SIRT1, respectively, suggesting that essential oils may positively influence liver antioxidant activity via the SIRT1/PGC-1α signaling pathway. In the present study, supplementation with both PEO and CEO reversed the downregulation of Prx3 and Trx2. Furthermore, the diet supplemented with CEO significantly upregulated the mRNA expression of SIRT1, PGC-1α, Nrf1, and Trx-R2 in LPS-challenged piglets. Further determination of the expression of key proteins in the mitochondrial function signaling pathway revealed that diet supplemented with PEO and CEO significantly upregulated the relative protein expression of SIRT1 and PGC1-α in the liver of LPS-challenged piglets. The above data indicate that the diet supplemented with PEO and CEO may increase mitochondrial biosynthesis and activity through the SIRT1/PGC1-α signaling pathway. The mRNA expression of PGC-1α and Trx2 in the LPS+CEO group was higher than that in the LPS+PEO group, suggesting that CEO has stronger mitochondrial antioxidant function, which may be due to the encapsulation technology protecting the essential oil from enzymatic degradation, allowing it to reach the small intestine for absorption, thereby improving bioavailability.

## CONCLUSION

In summary, this study demonstrated that both PEO and CEO can improve the growth performance of nursery piglets, reduce liver damage, enhance immune function by increasing immunoglobulin content and reducing inflammatory factor concentrations. Moreover, these supplements enhance antioxidant capacity by increasing antioxidant enzyme activity, antioxidant-related gene mRNA expression, and mitochondrial function key protein expression. The above results show that the addition of PEO and CEO to the diet has the therapeutic potential to reduce LPS-induced liver oxidative damage in nursery piglets by regulating the SIRT1/PGC-1α signaling pathway, and the improvement effect of CEO is better than that of PEO.

## Figures and Tables

**Table 1:** Ingredient and analyzed chemical compositions of control diets (as-fed basis)

Item	Content
Ingredient (%)	
Corn	57.70
Soybean meal (CP 46%)	12.50
Swelling corn	8.00
Full-fat soybean	8.00
Fermented soybean meal	4.00
Whey powder	3.00
Fish meal (CP 67%)	3.00
Calcium hydrogen phosphate	1.80
Limestone powder	0.50
*L*-Lysine (98%)	0.30
*L*-Threonine (98%)	0.10
*DL*-Methionine (98%)	0.08
Wheat middling	0.02
Premix^1)^	1
Total	100
Analyzed composition (%)	
Digestive energy (MJ/kg)	14.04
Crude protein	18.36
Lysine	1.28
Methionine	0.45
Methionine+cystine	0.72
Threonine	0.83
Calcium	0.81
Total phosphorus	0.69

1) Premix provided per kilogram of diet: Vitamin A, 13,000 IU; Vitamin D_3_, 3,100 IU; α-Tocopherol, 55 mg; Vitamin K_3_, 4.2 mg; Vitamin B_1_, 5 mg; Vitamin B_2_, 13 mg; Vitamin B_6_, 8 mg; Vitamin B_12_, 0.07 mg; Niacin acid, 35 mg; Pantothenic acid, 17 mg; Folic acid, 0.3 mg; Biotin, 0.08 mg; Choline chloride, 500 mg; Fe (FeSO_4_·H_2_O), 130 mg; Cu (CuSO_4_·5H_2_O) 10 mg; Zn (ZnO), 150 mg; I (KIO_3_), 0.4 mg; Mn (MnSO_4_·H_2_O), 8 mg; Se (Na_2_SeO_3_), 0.5 mg.

**Table 2:** Primer sequences for fluorescent quantitative PCR

Gene names	Forward primer (5’→3’)	Reverse primer (5’→3’)	Accession number
*CAT*	AGCTTTGCCCTTGCACAAAC	TCCATATCCGTTCATGTGCCTGTG	NM_214301.2
*SOD1*	AAGGCCGTGTGTGTGCTGAA	GATCACCTTCAGCCAGTCCTTT	NM_001190422.1
*SOD2*	GGCCTACGTGAACAACCTGA	TGATTGATGTGGCCTCCACC	NM_214127.2
*GSH-Px*	CCTCAAGTACGTCCGACCAG	GTGAGCATTTGCGCCATTCA	NM_214201.1
*SIRT1*	GGCAGGAGAAGGAAACAATGG	GTCGTCGTCGTCGTAGAAGTC	NM_001145750.2
*PGC-1α*	ACAACACGGACAGAACTGAGG	GCATCACAGGTATAACGGTAGGT	NM_213963.2
*Nrf1*	TATTCTGCTGTGGCTGATGGAGAG	GCTTGCGTTGTCTGGATGGTC	XM_021079000.1
*TFAM*	TCCTCCTCCTTCGTCGTAGTC	AGGCACTATGGGAAATCAGTCAAC	NM_001130211.1
*Nrf2*	GCCCCTGGAAGCGTTAAAC	GGACTGTATCCCCAGAAGGTTGT	XM_005671981.3
*Keap1*	TGCACGCTGCGATGGAG	GGGGTTCCAGATGACAAGGG	NM_001114671.1
*HO-1*	AGCACTCACAGCCCAACAG	GTACAAGGACGCCATCACC	NM_001004027.1
*NQO1*	TGCTTACACATACGCTGCCA	CGTGGATACCCTGCAGAGAG	NM_001159613.1
*Trx-R2*	GCACCTGCGTGAATGTTG	GCCCTCCAGTAGCGATGA	NM_001168702.1
*Trx2*	GACAGAAGTGCCCTTGA	AGCCATCTCCCAGCAAC	NM_001243705.1
*Prx3*	TATCCGACATGTGAGTGCCG	CCACAGCACACTTGTCAAGC	NM_001244531.1
*β-actin*	CTACACCGCTACCAGTTCGC	TAGGAGTCCTTCTGGCCCAT	XM_021086047.1

PCR, polymerase chain reaction; *CAT*, catalase; *SOD1*, superoxide dismutase 1; *SOD2*, superoxide dismutase 2; *GSH-Px*, glutathione peroxidase; *SIRT1*, silent information regulator 1; *PGC-1α*, peroxisome proliferator-activated receptor γ coactivator 1α; *Nrf1*, nuclear factor erythroid 2-related factor 1; *TFAM*, mitochondrial transcription factor A; *Nrf2*, nuclear factor E2-related factor 2; *Keap1*, Kelch-like ECH-associated protein 1; *HO-1*, heme oxygenase 1; *NQO1*, NAD(P)H quinone oxidoreductase 1; *Trx-R2*, thioredoxin reductase 2; *Trx2*, thioredoxin 2; *Prx3*, peroxiredoxin 3.

**Table 3:** The effect of dietary supplementation with plant essential oil (PEO) and coated plant essential oil (CEO) on growth performance of nursery piglets

Item	Treatment groups				SEM	p-value

	CON	LPS	LPS+PEO	LPS+CEO		
BW 21 d (kg)	6.83	6.80	6.82	6.80	0.03	0.979
BW 49 d (kg)	16.45^c^	16.53^c^	17.62^b^	18.92^a^	0.22	<0.001
ADG (g)	343.45^c^	347.62^c^	385.72^b^	426.79^a^	7.11	<0.001
ADFI (g)	533.33^c^	540.17^bc^	562.27^ab^	577.00^a^	5.07	0.002
FCR (g/g)	1.55^a^	1.56^a^	1.46^b^	1.35^c^	0.02	<0.001

CON, control group, piglets fed the basal diet and injected with 100 μg/kg BW saline; LPS, piglets fed the basal diet and injected with LPS at a dose of 100 μg/kg body weight; LPS+PEO, piglets fed the basal diet supplemented with 500 mg/kg PEO and injected with LPS; LPS+CEO, piglets fed the basal diet supplemented with 500 mg/kg CEO and injected with LPS.

a–c Means in the same row with different superscript differ significantly (p<0.05).

LPS, lipopolysaccharide; BW, body weight; ADG, average daily gain, ADFI, average daily feed intake; FCR, feed conversion ratio.

**Table 4:** The effect of dietary supplementation with plant essential oil (PEO) and coated plant essential oil (CEO) on the immune function and antioxidant activity in the liver of nursery piglets challenged by lipopolysaccharide (LPS)

Item	Treatment groups				SEM	p-value

	CON	LPS	LPS+PEO	LPS+CEO		
Immune function						
IgA (mg/g protein)	21.07^a^	13.04^d^	15.07^c^	18.02^b^	0.67	<0.001
IgG (mg/g protein)	79.64^a^	60.35^c^	65.93^b^	70.43^b^	1.63	<0.001
IgM (mg/g protein)	21.05^a^	13.07^d^	15.80^c^	18.07^b^	0.67	<0.001
IL-1β (ng/g protein)	39.20^b^	51.23^a^	46.11^ab^	44.47^b^	1.46	0.008
IL-6 (μg/g protein)	9.64^c^	12.35^a^	11.50^ab^	10.37^bc^	0.27	<0.001
TNF-α (μg/g protein)	0.74^c^	0.90^a^	0.85^ab^	0.78^b^	0.02	<0.001
Antioxidant activity						
T-AOC (U/mg protein)	9.27^a^	6.55^b^	7.05^b^	8.51^a^	0.27	<0.001
T-SOD (U/mg protein)	12.69^a^	9.31^c^	9.97^bc^	11.42^ab^	0.34	<0.001
GSH-Px (U/mg protein)	90.37^a^	72.45^c^	77.84^bc^	83.43^ab^	1.63	<0.001
CAT (U/mg protein)	8.64^a^	6.30^c^	7.01^bc^	7.84^ab^	0.22	<0.001
MDA (μmol/g protein)	0.72^c^	0.88^a^	0.81^ab^	0.75^bc^	0.02	<0.001

CON, control group, piglets fed the basal diet and injected with 100 μg/kg BW saline; LPS, piglets fed the basal diet and injected with LPS at a dose of 100 μg/kg body weight; LPS+PEO, piglets fed the basal diet supplemented with 500 mg/kg PEO and injected with LPS; LPS+CEO, piglets fed the basal diet supplemented with 500 mg/kg CEO and injected with LPS.

a–c Means in the same row with different superscript differ significantly (p<0.05).

IgA, immunoglobulin A; IgG, immunoglobulin G; IgM, immunoglobulin M; IL-1β, interleukin-1β; TNF-α, tumor necrosis factor-α; IL-6, interleukin-6; T-AOC, total antioxidant capacity; T-SOD, total superoxide dismutase; GSH-Px, glutathione peroxidase; CAT, catalase; MDA, malondialdehyde.

**Table 5:** The effect of dietary supplementation with plant essential oil (PEO) and coated plant essential oil (CEO) on the relative mRNA expression levels to the control of 1.0 in the liver of nursery piglets challenged by lipopolysaccharide (LPS)

Item	Treatment groups				SEM	p-value

	CON	LPS	LPS+PEO	LPS+CEO		
Antioxidant related genes						
*Nrf2*	1.00^b^	0.63^b^	1.14^ab^	1.66^a^	0.12	0.011
*Keap1*	1.00^a^	0.27^b^	0.25^b^	0.32^b^	0.07	<0.001
*HO-1*	1.00^c^	0.43^d^	1.76^a^	1.43^b^	0.11	<0.001
*NQO1*	1.00^b^	0.53^b^	2.11^a^	2.37^a^	0.23	0.003
*SOD1*	1.00^a^	0.38^b^	0.69^ab^	0.87^ab^	0.08	0.014
*SOD2*	1.00^ab^	0.34^c^	0.73^bc^	1.16^a^	0.08	<0.001
*CAT*	1.00^a^	0.29^b^	0.58^ab^	0.96^a^	0.09	0.007
*GSH-Px*	1.00	0.71	0.91	1.32	0.09	0.129
Mitochondrial function related genes						
*SIRT1*	1.00^a^	0.42^b^	0.73^ab^	1.00^a^	0.08	0.012
*PCG-1α*	1.00^ab^	0.41^c^	0.63^bc^	1.36^a^	0.10	<0.001
*Nrf1*	1.00^ab^	0.60^b^	0.85^ab^	1.36^a^	0.09	0.006
*TFAM*	1.00^a^	0.51^b^	0.63^ab^	0.90^ab^	0.07	0.027
*Prx3*	1.00^ab^	0.66^b^	1.42^a^	1.48^a^	0.10	0.005
*Trx-R2*	1.00^ab^	0.63^b^	1.19^ab^	1.41^a^	0.11	0.083
*Trx2*	1.00^c^	0.64^d^	1.30^b^	1.89^a^	0.10	<0.001

CON, control group, piglets fed the basal diet and injected with 100 μg/kg BW saline; LPS, piglets fed the basal diet and injected with LPS at a dose of 100 μg/kg body weight; LPS+PEO, piglets fed the basal diet supplemented with 500 mg/kg PEO and injected with LPS; LPS+CEO, piglets fed the basal diet supplemented with 500 mg/kg CEO and injected with LPS.

a–d Means in the same row with different superscript differ significantly (p<0.05).

*Nrf2*, nuclear factor E2-related factor 2; *Keap1*, Kelch-like ECH-associated protein 1; *HO-1*, heme oxygenase 1; *NQO1*, NAD(P)H quinone oxidoreductase 1; *SOD1*, superoxide dismutase 1; *SOD2*, superoxide dismutase 2; *CAT*, catalase; *GSH-Px*, glutathione peroxidase; *SIRT1*, silent information regulator 1; *PGC-1α*, peroxisome proliferator-activated receptor γ coactivator 1α; *Nrf1*, nuclear factor erythroid 2-related factor 1; *TFAM*, mitochondrial transcription factor A; *Prx3*, peroxiredoxin 3; *Trx-R2*, thioredoxin reductase 2; *Trx2*, thioredoxin 2.
